# Moralized, Marginalized and Sanitized: How Stigma Shapes STI Vaccination Development, Policy and Uptake

**DOI:** 10.1002/jia2.70138

**Published:** 2026-07-24

**Authors:** Charlie Firth, Susanne H. Hodgson

**Affiliations:** ^1^ Department of Paediatrics Oxford Vaccine Group University of Oxford Oxford UK; ^2^ NIHR Oxford Biomedical Research Centre Oxford UK; ^3^ The Jenner Institute Nuffield Department of Medicine University of Oxford Oxford UK

1

Vaccination has the potential to transform the prevention of sexually transmitted infections (STIs). In the current global context of rising incidence of STIs, growing antimicrobial resistance and limited resources for diagnosis and treatment of STIs in many global settings, vaccines for STIs could be a cost‐effective and impactful public health intervention.

For many infectious diseases, evidence of safety, efficacy and cost‐effectiveness is sufficient to support vaccine introduction in most settings. However, for STIs, the factors influencing vaccine development and implementation are more complex and nuanced. Stigma, societal perceptions, political priorities and moral frameworks exert a greater influence over STI vaccines than those targeting infectious diseases with less stigmatized transmission routes.

Stigma is multifaceted, comprising of labelling, stereotyping, separation, status loss and discrimination, which operate within wider systems of power [1]. It manifests through micro‐level mechanisms, such as perceived or experienced discrimination, as well as macro and structural‐level processes, where stigma shapes the unequal distribution of resources across sectors, including healthcare, housing and employment [2, 3]. In the context of STIs, this includes moralizing assumptions about sexual behaviour, blame and risk, and use of emotive terminology (“dirty” vs. “clean”). This stigma often manifests as delayed testing, non‐disclosure to partners, and reduced engagement with prevention and treatment services due to fear of judgement and social repercussions [4]. Stigma reinforces social and health disparities and influences prioritization of vaccine development, immunization policy and an individual's likelihood of seeking vaccination.

Vaccine hesitancy represents a growing challenge that limits the impact of even highly efficacious vaccines. The uptake of vaccines depends not only on individuals’ willingness but also on collective acceptance within families and communities. STI vaccines inherit the stigma associated with the infections they target, the communities they aim to protect, and the institutions promoting and delivering vaccination [5]. In contexts where sex is stigmatized or discussing it is taboo, vaccination could be interpreted as condoning or pre‐empting sexual behaviour, generating resistance even when the health benefits are clear [6]. Such dynamics of stigma are evident in practice, as illustrated by the differing ways HIV, human papillomavirus (HPV) and Mpox interventions have been framed, prioritized and funded within public health responses.

Stigma was central to the early moralization of HIV, as the disease was associated with already marginalized groups and framed as a consequence of their “deviant” behaviour. Terms such as “gay plague” and “gay cancer” legitimized and justified the discriminatory responses against those living with HIV [7]. This moral framing shaped institutional action too: the concentration of HIV among marginalized and stigmatized groups contributed to a delayed and underfunded response, indicative of limited political urgency. Moralized and politicized understanding of HIV in the United States and other countries shaped funding resources, preferentially supporting abstinence‐focused programmes over more effective evidence‐based prevention strategies [8]. As a result, stigma functioned as an overarching force in public health decision‐making, resulting in delayed public health interventions, inefficient use of resources, and persistent barriers to equitable access to care and treatment.

The implementation of HPV vaccination illustrates how stigma can be actively negotiated and strategically managed in public health communication. Despite HPV being an STI associated with cervical, anal and oropharyngeal cancers, vaccination programmes, particularly those targeting adolescents, have purposefully “desexualized” HPV by obscuring its sexual mode of transmission. Instead, the HPV vaccine has been “sanitized” and framed predominantly as an anti‐cancer intervention [9]. While this reframing has been effective in reducing moral stigma and increasing acceptability for the vaccine among young people and their families, it also illustrates the enduring influence of stigma in shaping public health narratives, with sexual aspects of disease downplayed to avoid controversy and resistance.

Mpox outbreaks highlight how intersectionality shapes public health, impacting both perception and response. During the 2022 outbreak in the UK and the United States, the concentration of Mpox cases among gay and bisexual men raised concerns about reinforcing existing and historical stigma [10]. In contrast, in many African contexts, Mpox has historically been framed as a general infectious disease rather than a sexually stigmatized condition, exemplifying that stigma is not inherent to diseases but socially constructed [11]. Although Mpox is not strictly an STI, the delivery of Mpox vaccines through sexual health clinics in the UK and the United States risked reinforcing a negative association with sexual behaviour that could discourage engagement with care. At the same time in the United States, the burden of severe disease disproportionately affected Black men, illustrating how stigma intersects with racial inequality to amplify health inequalities. The decision by the World Health Organization to rename “monkeypox” as Mpox reflected an institutional recognition of the racialized and colonial connotations embedded in disease nomenclature, and how stigma can be reproduced and contested through language [12]. Adoption of the name Mpox has been more common in locations with greater LGBTQ acceptance and lower in locations governed by leaders who had recently propagated infectious disease‐related misinformation. Indeed, the US Health and Human services decision to revert to using the term “monkey pox” in direct contradiction to affected communities wishes, exemplifies the power of language to reinforce stigma [13].

Ongoing geopolitical instability and conflict are prompting governments to prioritize defence spending, constraining funding for global public health interventions. In this context, vaccines for conditions framed as non‐fatal, politically sensitive or attributable to individual responsibility are likely to be de‐prioritized and access limited, particularly in low‐ and middle‐income settings where health systems are subject to shifting donor priorities.

In parallel, the governance of vaccination is becoming increasingly politicized. Recent changes in leadership and immunization recommendations signal growing tensions between scientific expertise and political influence [14]. The re‐emergence of discredited vaccine safety claims and challenges to established vaccination practices undermine public confidence and illustrate how stigma and misinformation can be mobilized within broader ideological agendas [15]. These shifts extend beyond geographical borders, contributing to the global circulation of anti‐science narratives and weakening confidence in evidence‐based policy.

Within this sobering landscape, STI vaccines occupy a particularly vulnerable position. Their association with stigma, combined with reduced public funding and political sensitivity, diminishes both market incentives and policy prioritization. As a result, scientific advances alone are insufficient. There is a need to confront the structural, social and political dimensions of stigma, before equitable and successful development, implementation and uptake of STI vaccines can be realized. Addressing stigma must, therefore, be central, not peripheral, to the future of STI prevention.

## Author Contributions

*Conceptualization*: CF and SHH. *Writing – original draft*: CF and SHH. *Writing – review and editing*: CF and SHH.

## Conflicts of Interest

The authors declare no conflicts of interest.

## Data Availability

Not applicable.
